# Intramuscular Hydatid Cyst of the Thigh: A Rare Presentation in an Elderly Patient

**DOI:** 10.7759/cureus.68504

**Published:** 2024-09-03

**Authors:** Shruthi Bikkumalla, Imran Ali Khan, Sanjeev G Gianchandani Gyani, Anup A Zade, Suresh R Chandak, Sai Goutham Rekavari, Aditya S Pedaprolu, Yashraj Jain

**Affiliations:** 1 General Surgery, Jawaharlal Nehru Medical College, Datta Meghe Institute of Higher Education and Research, Wardha, IND; 2 Minimal Access and Robotic Surgery, Jawaharlal Nehru Medical College, Datta Meghe Institute of Higher Education and Research, Wardha, IND

**Keywords:** differential diagnosis, surgical excision, thigh swelling, echinococcosis, intramuscular hydatid cyst

## Abstract

Intramuscular hydatid cysts are an uncommon presentation of echinococcosis, with most cases occurring in the liver and lungs. This case report describes an 81-year-old male who presented with a painless, progressively enlarging swelling in the right thigh noticed over the past year. The patient's history included trauma to the area from an animal-related incident five years earlier. Physical examination revealed a firm, irregular, and non-tender mass. Ultrasonography suggested a multiloculated cystic lesion with calcification, raising suspicions of a hematoma or hydatid cyst. Fine-needle aspiration cytology indicated an organized hematoma with secondary calcification. Given the diagnostic uncertainty, surgical excision revealed a 3x3 cm hydatid cyst in the intramuscular plane. A histopathological examination confirmed the diagnosis. The patient's postoperative course was uneventful, with no signs of recurrence at the three-month follow-up. This case underscores the importance of considering hydatid disease in the differential diagnosis of intramuscular swellings, particularly in patients with relevant exposure histories.

## Introduction

Hydatid disease, also known as echinococcosis, is a parasitic infection caused by the larval stages of *Echinococcus* species, most commonly *E. granulosus* and *E. multilocularis*. It is a significant public health concern in endemic regions, including parts of South America, the Middle East, and Eastern Europe, where livestock farming and close contact with domestic dogs facilitate transmission [1]. Humans are accidental intermediate hosts, acquiring the infection through ingestion of eggs shed in the feces of definitive hosts, such as dogs [2].

The liver and lungs are the most common sites of hydatid cysts, accounting for approximately 75% and 15% of cases, respectively [3]. In contrast, primary involvement of the musculoskeletal system is rare, representing only 2-3% of cases [4]. Among these, intramuscular hydatid cysts are exceptionally uncommon, with only a few reported cases in the literature. The rarity of this presentation can lead to diagnostic challenges, as the clinical and radiological findings may mimic other conditions, such as abscesses, hematomas, or soft tissue tumors [5].

Clinical manifestations of intramuscular hydatid cysts are often nonspecific, typically presenting as painless, slow-growing masses. The diagnosis is often made incidentally or during investigation for unrelated symptoms. Imaging studies, such as ultrasound, computed tomography (CT), or magnetic resonance imaging (MRI), are crucial for identifying cystic lesions and suggesting a diagnosis. However, definitive diagnosis often requires histopathological confirmation, as serological tests may not always be reliable due to low sensitivity [6]. This case report describes an unusual presentation of an intramuscular hydatid cyst in an elderly patient with a history of animal-related trauma. The case highlights the importance of considering hydatid disease in the differential diagnosis of atypical soft tissue masses, especially in endemic regions or individuals with relevant exposure histories.

## Case presentation

An 81-year-old male presented to our clinic with a progressively enlarging swelling in the right thigh, which had been asymptomatic. The patient reported noticing the swelling one year prior, with a gradual increase in size over time. He denied any associated pain, fever, or weight loss. Notably, the patient had a history of trauma to the affected area caused by an animal-related incident five years earlier, but no immediate complications were reported at that time. On physical examination, the patient was found to have soft to cystic, non-tender, irregular margins with fixed underlying muscles. The swelling measures approximately 3x3 cm over the vastus lateralis muscle of the right thigh. The overlying skin appeared normal, with no signs of erythema infection or dilated veins, and no inguinal lymph nodes palpable. The rest of the physical examination was unremarkable.

**Figure 1:**
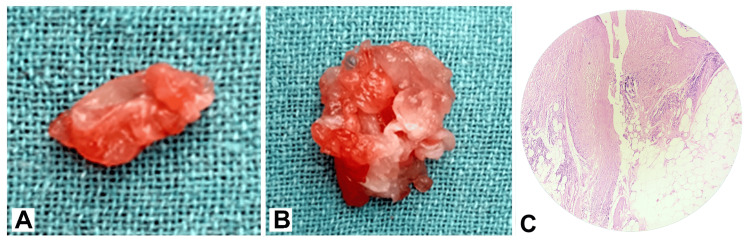
(A, B) A 3x3 cm hydatid cyst was identified within the intramuscular plane during the operation, showing daughter cysts resembling echinococcus. (C) The hydatid cyst wall is shown.

Initial diagnostic imaging with ultrasonography (USG) revealed a multiloculated, well-defined cystic lesion with foci of calcification in the intramuscular plane, suggesting a differential diagnosis of either a hematoma or a hydatid cyst. Fine-needle aspiration cytology (FNAC) was performed to investigate further, revealing cytomorphological features consistent with an old, organized hematoma with secondary calcification. USG abdomen, pelvis, and thorax were done to rule out any other cystic lesions. Despite the non-specific findings, the possibility of a hydatid cyst could not be excluded based on the patient's clinical history and imaging characteristics. Given the diagnostic uncertainty and potential complications associated with hydatid disease, the patient underwent surgical excision of the cystic swelling. During the operation, a 3x3 cm hydatid cyst was identified within the intramuscular plane, showing daughter cysts resembling *Echinococcus* (Figures 1A-1B). The cyst was carefully excised, followed by a thorough wash with a scolicidal agent, 5% cetrimide, to minimize the risk of spillage and subsequent anaphylaxis or dissemination. The excised specimen was sent for histopathological examination, confirming a hydatid cyst diagnosis (Figure 1C).

Postoperatively, the patient's recovery was uneventful. He was discharged with tablet albendazole 400 mg bd for four-week cycles repeated three times, separated by 14-day intervals (albendazole treatment of human cystic echinococcosis), and he was also given instructions for follow-up to monitor for any recurrence or complications. At the three-month follow-up, the patient reported no complaints, and clinical examination revealed no evidence of recurrence. This case highlights the importance of considering hydatid cysts in the differential diagnosis of intramuscular swellings, particularly in regions where echinococcosis is endemic or in patients with a relevant history of animal exposure.

## Discussion

Hydatid disease, caused by the larval stage of the *Echinococcus* tapeworm, is a zoonotic infection that primarily affects the liver and lungs. However, in rare cases, the cysts can develop in other organs and tissues, including skeletal muscles. Intramuscular hydatid cysts represent only 2-3% of all cases of hydatid disease, making this presentation unusual and diagnostically challenging [5,7]. The clinical presentation of intramuscular hydatid cysts is often non-specific. Patients may present with a slowly growing, painless mass, as was observed in this case. The lack of symptoms and the condition's rarity can delay diagnosis, often leading to misdiagnosing other conditions such as soft tissue tumours, abscesses, or hematomas [8]. In our case, the patient's history of trauma and the imaging findings initially suggested a hematoma, which was further supported by cytological examination. However, the possibility of a hydatid cyst was considered due to the presence of calcifications on imaging and the patient's relevant exposure history.

USG is a valuable initial imaging modality for evaluating cystic lesions, as it can reveal the cyst's morphology and the presence of daughter cysts, characteristic of hydatid disease [9]. However, USG findings were non-specific in this case, necessitating further investigation. FNAC was performed but did not yield definitive results, highlighting the limitations of cytological evaluation in diagnosing hydatid disease. Surgical excision remains the treatment for hydatid cysts, particularly in cases where the cyst is localized and accessible [10]. In this patient, complete cyst excision was achieved, and thorough cetrimide irrigation was used intraoperatively to minimize the risk of cyst rupture and subsequent anaphylaxis or dissemination. Histopathological examination confirmed the diagnosis of a hydatid cyst, characterized by a laminated membrane and protoscolices.

Postoperative follow-up is crucial to monitor for recurrence, as hydatid disease can recur if the cyst contents are not completely removed or if spillage occurs during surgery [11]. In this case, the patient had an uneventful recovery; no recurrence was noted during the three-month follow-up. This outcome emphasizes the importance of meticulous surgical technique and postoperative care. This case highlights the need for a high index of suspicion for hydatid disease in patients presenting with intramuscular cystic lesions, especially in endemic areas or in individuals with relevant exposure histories. It also underscores the importance of combining clinical, radiological, and histopathological findings to reach an accurate diagnosis.

## Conclusions

This case report highlights the rare occurrence of an intramuscular hydatid cyst in the thigh of an elderly patient, emphasizing the importance of considering hydatid disease in the differential diagnosis of asymptomatic swellings, especially in regions with endemic echinococcosis or in individuals with a history of exposure to animals. Despite its rarity and atypical presentation, a hydatid cyst should not be overlooked as a potential diagnosis. Timely surgical intervention with careful handling of the cyst and thorough cleansing is crucial to prevent complications such as anaphylaxis or dissemination. This case underscores the need for a high index of suspicion and comprehensive diagnostic evaluation in similar presentations to ensure appropriate management and favorable outcomes.
